# Postpartum extracorporeal membrane oxygenation of a woman with COVID-19-related acute respiratory distress syndrome: A case report

**DOI:** 10.1097/MD.0000000000026798

**Published:** 2021-07-30

**Authors:** Weizhao Huang, Zhou Cheng, Xiaozu Liao, Liqiang Wang, Junlin Wen, Jianwei Li, Haiming Jiang, Yong Yuan, Binfei Li

**Affiliations:** a Department of Cardiothoracic Surgery, Zhongshan People's Hospital (Zhongshan Hospital of Sun Yat-Sen University) China; b Department of Anesthesiology, Zhongshan People's Hospital (Zhongshan Hospital of Sun Yat-Sen University) China; c Intensive Care Unit, Zhongshan People's Hospital (Zhongshan Hospital of Sun Yat-Sen University) China; d Department of Cardiology, Zhongshan People's Hospital (Zhongshan Hospital of Sun Yat-Sen University), China.

**Keywords:** acute respiratory distress syndrome, case report, COVID-19, extracorporeal membrane oxygenation, postpartum

## Abstract

**Introduction:**

Patients with coronavirus disease (COVID-19) may develop acute respiratory distress syndrome (ARDS). There have been few reports of postpartum woman with ARDS secondary to COVID-19 who required respiratory support using veno-venous extracorporeal membrane oxygenation (ECMO). We present the case of a 31-year-old woman who was admitted to hospital at 35 weeks gestation with ARDS secondary to COVID-19 and required ECMO during the postpartum period.

**Patient concerns:**

The patient had obvious dyspnea, accompanied by chills and fever. Her dyspnea worsened and her arterial oxygen saturation decreased rapidly.

**Diagnosis:**

ARDS secondary to COVID-19.

**Interventions:**

Emergency bedside cesarean section. Medications included immunotherapy (thymosin α 1), antivirals (lopinavir/ritonavir and ribavirin), antibiotics (imipenem-cilastatin sodium and vancomycin), and methylprednisolone. Ventilatory support was provided using invasive mechanical ventilation. This was replaced by venous-venous ECMO 5 days postpartum. ECMO management focused on blood volume control, coagulation function adjustment, and airway management.

**Outcomes:**

The patient was successfully weaned for ECMO and the ventilator and made a good recovery.

**Conclusion:**

Special care, including blood volume control, coagulation function adjustment, and airway management, should be provided to postpartum patients with ARDS secondary to COVID-19 who require ECMO support.

## 1 Introduction

The coronavirus disease (COVID-19) pandemic, started in Wuhan, China, at the end of 2019, and spread worldwide. According to the World Health Organization, the number of cases continues to rise, and 2.3% of patients become critically ill and require invasive mechanical ventilation.^[1]^ Extracorporeal membrane oxygenation (ECMO) can be used to treat patients with extremely severe respiratory distress. COVID-19 has a 1.4–4.3% case fatality rate overall,^[1,2]^ which increases to 61.5% in critically ill adults.^[3]^ In China, according to clinical reports, the management of COVID-19 pneumonia using ECMO has not been effective to date.^[4,5]^ There are few reports of ECMO use for treatment of COVID-19-related acute respiratory distress syndrome (ARDS) from other countries. We report the case of a woman who developed COVID-19-related ARDS in late pregnancy and required ECMO support postpartum. ECMO management was complicated because the pathophysiological characteristics included COVID-19, ARDS, and postpartum status.

## 2 Case report

A 31-year-old woman with an uneventful third pregnancy was admitted to a local hospital in Zhongshan City in Guangdong Province in China in February 2020 at 35-weeks gestation, with a 4-day history of fever and sore throat, and a 3h history of progressive dyspnea. The patient had visited Xiaogan, a city near Wuhan, two weeks before the onset of her symptoms.

The patient had obvious dyspnea, accompanied by chills and fever, with a body temperature of 39.3°C on admission. Chest computed tomography showed left lower lobe consolidation of the lungs. High-flow nasal cannula oxygen therapy was initiated (80% concentration, flow rate 50 L/min). However, her dyspnea rapidly worsened and her arterial oxygen saturation decreased to 60%. Invasive mechanical ventilation was initiated and she underwent an emergency bedside cesarean section after careful consideration by the obstetricians and consent of the patient. Eight hours later a throat swab sample was taken and the Guangdong Centers for Disease Control confirmed the diagnosis of COVID-19 by real-time reverse transcription PCR assay.

She received invasive mechanical ventilation using synchronized intermittent mandatory ventilation (SIMV) mode with a peak inspiratory pressure (Pinsp) of 35 cm H_2_O, a positive end-expiratory pressure (PEEP) of 15 cm H_2_O, and fraction of inspired oxygen (FiO_2_) of 80%. Her arterial PO_2_ was 72 mmHg, PCO_2_ 41 mmHg, and arterial oxygen saturation was 93%. Her body temperature was 36.3°C, her heart rate was 160 bpm, and her respiratory rate was 25 breaths/min. Her blood pressure was 122/79 mmHg on noradrenaline 0.3 μg/kg/min.

**Figure 1:**
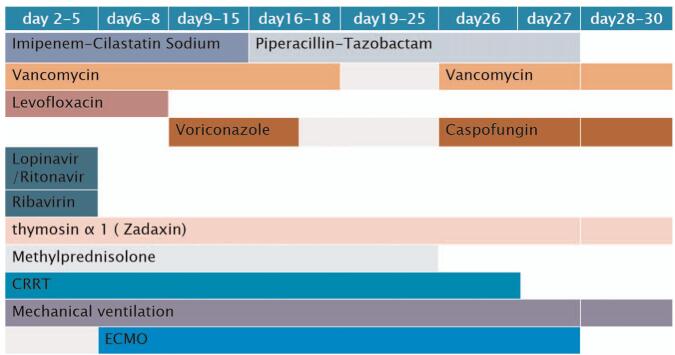
The medication and the therapies for the case in timeline. CRRT = continuous renal replacement therapy, ECMO = extracorporeal membrane oxygenation.

She was given thymosin α 1 (1.6 mg daily, subcutaneous injection) as immunotherapy; lopinavir/ritonavir (500 mg twice daily, orally) and ribavirin as antiviral therapy; imipenem-cilastatin sodium, and vancomycin as antibiotic therapy; and methylprednisolone (80 mg daily, intravenously) to reduce lung inflammation. She developed fluid overload which was controlled by diuretics and limiting her fluid intake (Fig. 1).

After this treatment, her oxygenation and circulation improved, but on day 5 of her illness her arterial PO_2_ dropped to 61 mmHg, her PCO_2_ increased to 40 mmHg, and her arterial oxygen saturation dropped to 90% on SIMV (Pinsp 35 cm H_2_O, PEEP 16 cm H_2_O, FiO_2_ 100%).

This deterioration led us to initiate venous-venous ECMO (Medtronic Inc.). We inserted the outflow cannula through right femoral vein and the inflow cannula through right jugular vein. We chose a 25F outflow cannula (Medtronic Inc.) and 17F inflow cannula, according to the patient's body surface area. Initiation of ECMO resulted in an immediate improvement in oxygenation, and lung-protective ventilation was initiated.

Heparin was used for anticoagulation during ECMO support, and the patient's target activated clotting time (ACT) was 180–220 s. Her D-dimer levels, prothrombin time, activated partial thromboplastin time, fibrinogen, and platelets were monitored.

**Figure 2:**
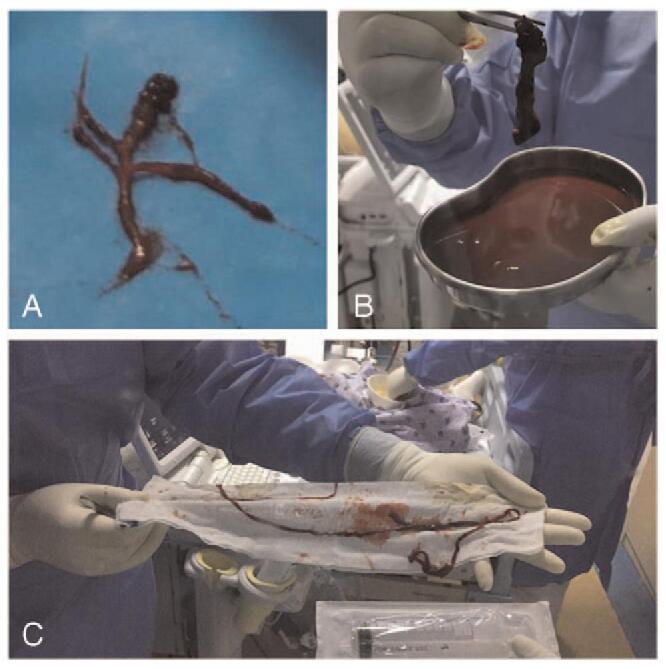
Blood clots in the bronchi and extracorporeal membrane oxygenation (ECMO) inflow cannula. (A). Blood clots were removed from the main bronchi and the peripheral bronchi by bronchoscopy. (B&C). Blood clots removed from the ECMO inflow cannula.

Considering the need for better tolerance and airway management, percutaneous tracheostomy was performed on day 18. Although heparin was stopped before the surgery, and ACT was maintained for 130 s, severe intratracheal hemorrhage occurred after the surgery. A second operation was required to stop the bleeding. After repeatedly removing blood clots under bronchoscopy, the tidal volume gradually increased from 100 mL to 350 mL 48 h later. On day 25, the ECMO flow suddenly decreased to 0 L/min, and the pressure increased. Multiple blood clots were found in the inflow cannula (Fig. 2). The ventilator parameters were adjusted, and the ECMO circuit and cannula were replaced.

**Figure 3:**
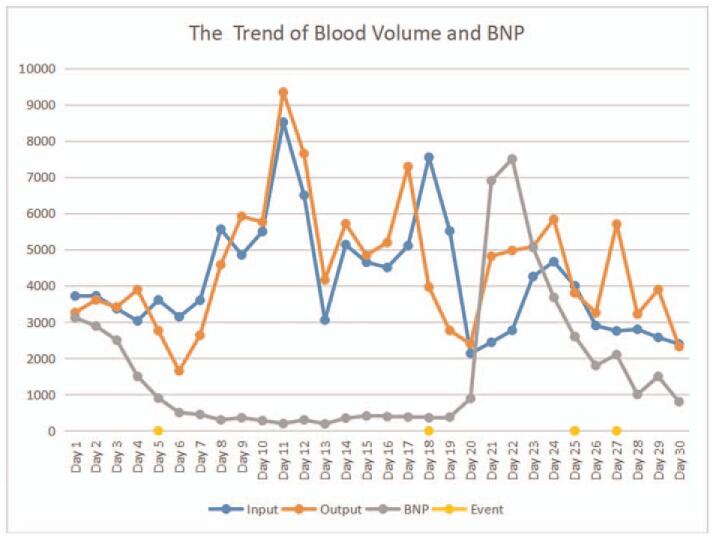
Relationship between blood volume and B-type natriuretic peptide levels during the patient's hospitalization for COVID-19. Events: Day 5, extracorporeal membrane oxygenation (ECMO) support started; Day 18, percutaneous tracheostomy was performed and severe intratracheal hemorrhage occurred; Day 25, thrombosis was found in the inflow cannula; Day 27, ECMO was removed.

**Figure 4:**
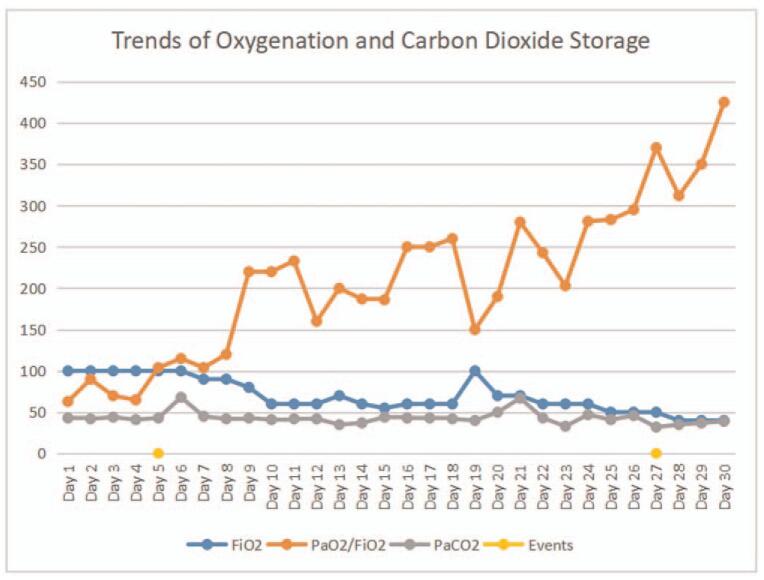
Trends in blood oxygenation and carbon dioxide levels during the patient's hospitalization for COVID-19. Events: On Day 5, extracorporeal membrane oxygenation (ECMO) support was initiated; on Day 27 ECMO was stopped.

After the patient's condition stabilized, PEEP was gradually down regulated, and the fluid balance became negative (Fig. 3). On day 27, a chest X-ray revealed resorption of the ground-glass opacities in both lungs, and the blood gas analysis revealed that PO_2_/FiO_2_ was 370 mmHg and PCO_2_ was 36 mmHg with an ECMO flow of 2.0 L/min, (Fig. 4), and so the ECMO was successfully withdrawn. On day 38, the patient was successfully weaned from the ventilator and subsequently made a good recovery.

## 3 Discussion

Since the start of the COVID-19 pandemic, there have been many critical cases of COVID-19 in China that were treated with ECMO. The case fatality rate is high.^[4,5]^ This case treatment process was complicated because the patient's pathophysiological characteristics included COVID-19, ARDS, and postpartum status. This report focuses on the ECMO management.

Due to the physiological hypercoagulability postpartum,^[6,7]^ in order to achieve the target ACT value, we used a relatively large initial dose of heparin (approximately 12 U/kg). The ACT increased to 170 s, resulting in bleeding at multiple puncture points, which made it difficult to achieve hemostasis during the surgery. When the ACT decreased to 140 s, a thrombus developed in the pipeline and the pump stopped. Hemorrhage and embolism can have severe consequences in critical patients. For anticoagulation management, the ACT safety range is relatively narrow (150–160 s), which may be related to the physiological characteristics of pregnant women. The management of anticoagulant intensity should not only follow the guidelines, but should also consider the specific condition of the patient, including comorbidities, and refer to the clinical bleeding and coagulation status when making decisions regarding clinical management.

During late pregnancy, there is a large volume of blood stored in tissue, most of which re-enters the circulation during the early postpartum period, resulting in parturient women having an increased blood volume. Additionally, the treatment of lung disease requires a low blood volume, therefore, strict liquid management measures were taken with this patient. A cumulative negative balance of more than 10 L developed over the course of 30 days, which not only ensured less lung exudation, but also reduced the cardiac load and tissue edema.

As observed in other severe COVID-19 patients, the main manifestations of COVID-19 in this patient were acute pulmonary inflammation and a decreased oxygenation index in the early stage, and decrease of the tidal volume and carbon dioxide storage in the later stage. The duration of the illness is about 20 days on average before the SARS-CoV-2 antigen tests revert to negative.^[8]^ As the infection resolves, acute pulmonary inflammation subsides, and oxygen saturation improves. The removal of ECMO depends on the correction of carbon dioxide storage. Histology of autopsy pulmonary tissue from a fatal case of COVID-related ARDS showed that there were multiple mucus emboli in the small airways and alveoli,^[9]^ which were difficult to remove via fibrobronchoscope. In addition, the increase in body mass index of parturient women and the elevation of diaphragm often lead to decreased tidal volume, which can reduce the diffusion of carbon dioxide. Through tracheotomy and autonomous respiration, diaphragm activity can be promoted, autonomously discharging phlegm, reducing mucus secretion, increasing tidal volume, and discharging carbon dioxide.

In conclusion, blood volume control, coagulation function adjustment, and airway management were important in the management of this rare and unusual case. In the context of the COVID-19 global pandemic, it provides insight into the specific considerations that are needed in the treatment of COVID-19-related ARDS during the postpartum period.

## Author contributions

**Conceptualization:** Weizhao Huang, Zhou Cheng, Jianwei Li, Haiming Jiang, Binfei Li, Xiaozu Liao.

**Data curation:** Weizhao Huang, Zhou Cheng, Liqiang Wang, Jianwei Li, Junlin Wen, Xiaozu Liao.

**Formal analysis:** Liqiang Wang, Yong Yuan.

**Funding acquisition:** Weizhao Huang, Haiming Jiang, Binfei Li.

**Investigation:** Weizhao Huang, Zhou Cheng, Haiming Jiang, Binfei Li.

**Methodology:** Yong Yuan, Binfei Li.

**Project administration:** Junlin Wen.

**Resources:** Liqiang Wang.

**Writing – original draft:** Weizhao Huang.

**Writing – review & editing:** Weizhao Huang.
